# Longitudinal analysis of quality of life in primary lung cancer patients with chlamydia pneumoniae infection: a time-to-deterioration model

**DOI:** 10.1186/s12890-024-02860-x

**Published:** 2024-01-17

**Authors:** Zishan Chen, Jinman Zhuang, Maolin Liu, Xinying Xu, Yuhang Liu, Shuyan Yang, Jinbao Xie, Nanlong Lin, Fancai Lai, Fei He

**Affiliations:** 1 Department of Epidemiology and Health Statistics, Fujian Provincial Key Laboratory of Environment Factors and CancerSchool of Public Health, Fujian Medical University, Fuzhou, 350122 Fujian Province China; 2 Department of Thoracic Surgery, the First Affiliated Hospital of Fujian Medical University, Fuzhou, China

**Keywords:** Primary lung cancer, Chlamydia pneumonia, Health-related quality of life, Time to deterioration

## Abstract

**Background:**

Chlamydia pneumoniae (Cpn) IgG and IgA has been strongly linked to lung cancer, but its impact on patients' quality of life remains unclear. Our objective was to investigate the relationship between pre-treatment Cpn IgG and IgA and time to deterioration (TTD) of the HRQoL in patients with primary lung cancer.

**Methods:**

A prospective hospital-based study was conducted from June 2017 to December 2018, enrolling 82 patients with primary lung cancer admitted to the First Affiliated Hospital of Fujian Medical University for questionnaire surveys. Cpn IgG and IgA was detected by microimmunofluorescence method. HRQoL was assessed at baseline and during follow-up using the EORTC Quality of Life Questionnaire version 3.0 (EORTC QLQ-C30) and EORTC Quality of Life Questionnaire-Lung Cancer (EORTC QLQ-LC13). HRQoL scores were calculated using the QoLR package, and TTD events were determined (minimum clinically significant difference = 5 points). Cox regression analysis was used to evaluate the effect of Cpn IgG and IgA on HRQoL.

**Results:**

We investigated the relationship between Cpn IgG and IgA and quality of life in patients with primary lung cancer. The study was found that 75.61% of cases were Cpn IgG + and 45.12% were Cpn IgA + . Cpn IgA + IgG + was 41.46%. For EORTC QLQ-C30, Physical function (PF) and Pain (PA) TTD events on the functional scale and Symptom scale were the most common during follow-up. After adjusting for gender and smoking status, Pre-treatment Cpn IgA + was found to signifcantly delay TTD of Physical functioning(*HR* = 0.539, *95% CI*: 0.291–0.996,* P* = 0.048). In addition, Cpn IgG + before treatment significantly delayed TTD in Emotional functioning (*HR* = 0.310, *95% CI*: 0.115–0.836, *P* = 0.021). For EORTC QLQ-LC13, deterioration of dyspnea (LC-DY) was the most common event. However, Cpn IgG and IgA before treatment had no effect on the TTD of EORTC QLQ-LC13 items.

**Conclusions:**

According to EORTC QLQ-C30 and EORTC QLQ-LC13, Cpn IgA delayed TTD in Physical functioning and Cpn IgG delayed TTD in Emotional functioning.

## Introduction

Lung cancer is a malignant tumor with the highest morbidity and mortality rate in the world [1]. Early diagnosis and treatment are crucial in improving the prognosis of patients with lung cancer. However, due to atypical early symptoms, a lack of effective detection methods and strong invasiveness, most patients with lung cancer are already in the middle or late stages by the time they seek medical attention, resulting in a poor prognosis [2]. With the development of diagnosis and treatment technology and the continuous research and development of new drugs, precision medicine has extended the survival time of lung cancer patients, especially the emergence and application of targeted drugs, so that more and more lung cancer patients' survival time has exceeded 5 years. At the same time, many cancer survivors experience health damage as they live longer [3]. Therefore, it is of great clinical significance to pay attention to and improve health-related symptoms in patients with primary lung cancer.

Health-related quality of life is a multidimensional concept that relates to an individual's general health status. It serves as nationally representative tool for examining the lifestyle characteristics of cancer survivors. It includes areas related to social functioning, emotional, mental and physical health that are impaired in cancer patients. Although patients' perceptions of life, satisfaction, and happiness tend to improve after undergoing early screening and treatment for cancer, many challenges persist for cancer survivors. These include long-term complications associated with treatment that can significantly impact a patient's health-related quality of life [4–6]. Studies have shown that occupational cancer patients have poorer health-related quality of life than the general population [7, 8].

At present, the pathogenesis of lung cancer remains incompletely understood. In addition to tobacco smoke inhalation, which has been established as a confirmed risk factor, respiratory diseases, diet, infection, occupational exposure and other factors may also be related to the occurrence of lung cancer [9]. Chlamydia pneumoniae (Cpn) is a pathogenic member of Chlamydia genus with human as the sole host. It can cause pneumonia or other respiratory diseases. However, the control of Cpn in practice is insufficient, as most infected individuals exhibit mild or no clinical symptoms [10]. Cpn is primarily transmitted through the respiratory tract and can elicit specific cellular and humoral immune responses Due to limited host immunity, persistent, insidious and recurrent infections are prone to occur, leading to chronic inflammatory stimulation that creats conditions conducive to tumor occurrence, spread and metastasis [11]. As such, controlling Cpn infection may have significant clinical implications for improving health-related symptoms in patients with lung cancer.

Studies have shown that Cpn infection may be associated with primary lung cancer [12], but no studies have investigated the relationship between Cpn infection and health-related quality of life (HRQoL) in patients with primary lung cancer. The time to deterioration (TTD) model is a longitudinal time-event analysis used to evaluate the change of HRQoL over time in cancer patients after treatment, which can solve the problem of missing HRQoL data in long-term follow-up [13–15]. In this prospective study, we aimed to analyze the relationship between Cpn infection before treatment and TTD in HRQoL in primary lung cancer survivors.

## Methods

### Study patients

This is a hospital-based prospective study conducted in the Department of Thoracic Surgery, the First Affiliated Hospital of Fujian Medical University. The inclusion criteria were as follows: (1) Confirmed by fibrobronchoscopy or histology; (2) The date of diagnosis was from June 2017 to December 2018; (3) New cases of primary lung cancer; (4) No restriction on gender, age and pathological stage of cases; (5) Serum samples for testing chlamydia infection status are available. Exclusion criteria:(1) Secondary lung cancer was confirmed by pathological diagnosis; (2) Lack of pathological diagnosis; (3) Those who cannot answer questions clearly. This study was approved by the Ethics Review Committee of Fujian Medical University, and all subjects signed informed consent.

### Collection of baseline information and sample

A structured questionnaire was designed for this study. Data was collected by trained investigators during face-to-face interviews with patients. Data were collected related to the following variables: general information (age, gender, education level, height and weight), smoking and drinking history, and baseline quality of life (QoL) scores. The data was collected when patients were admitted to hospital.

After all subjects signed the informed consent and before treatment, 5 ml of fasting peripheral venous blood was collected by non-anticoagulant vacuum blood collection by hospital nurses in the morning. The collected samples were processed immediately, centrifuged at 2000 rpm for 10 min, and the separated serum was placed in a -80℃ cryogenic refrigerator for storage and reserve to avoid repeated freeze–thaw. microimmunoflourescence (MIF) kit (Chlamydia IgGSeroFIATM kit and Chlamydia IgA SeroFIATM kit, Savyon, Israel) for detection of Chlamydia pneumoniae specific antibody IgG and IgA in serum.

### Health related Quality of life assessments

Quality of life was assessed at baseline and during follow-up using the European Organization for Research and Treatment of Cancer (EORTC) Quality of Life Questionnaire 3.0 (EORTC QLQ-C30) and the EORTC Quality of Life Questionnaire—Lung Cancer (EORTC QLQ-LC13). EORTC QLQ-C30 questionnaire comprises 30 items, categorized into 5 functional dimensions (physical functioning, role functioning, emotional functioning, cognitive functioning, social functioning), 9 symptom dimensions (fatigue, nausea and vomiting, pain, dyspnea, insomnia, appetite loss, constipation, diarrhea, financial difficulties), and 1 overall quality of life dimension [16]. Each item is scored on a scale from 1 to 4, and the average score for each dimension, known as the raw score (RS), is linearly transformed using a range-scaling method to obtain a standardized score (SS) on a percentage scale [17]. The SS for functional dimensions and the overall quality of life dimension is directly proportional to the score, indicating better functioning with higher scores. Conversely, the SS for symptom dimensions is inversely proportional to the score, signifying more severe symptoms and lower quality of life with higher scores.. The EORTC QLQLC13 questionnaire includes 13 questions to assess lung cancer-related symptoms, treatment-related side effects, and analgesic use [17]. A higher score in QLQ-C30-LC13 indicates a higher level of symptoms.

### Follow‑up

Survival time was defined as the time from admission to hospital (June 2017 to December 2018) to death or the end of follow-up on December 2, 2020. All patients were followed up every 3–6 months during the first year and annually thereafter.

### Time to deterioration model

TTD was defined as the duration from the patient's enrollment in the study to the initial occurrence of clinically significant deterioration, as measured against the baseline HRQoL scores using the corresponding assessment tools. [18]. Minimum clinically significant difference refers to the minimum difference in HRQoL scores considered to be clinically important; it is an important indicator to judge the clinical relevance of results [19]. In our study, TTD was defned as the time from the frst observation with defnitive deterioration with a > 5-point, and no subsequent observations with a < 5-point decrease compared to baseline in the EORTC QLQ-C30 and EORTC QLQ-LC13 [15].

### Statistical analysis

The QoLR package is used to calculate HRQoL scores and determine TTD events in EORTC QLQ-C30 and EORTC QLQ LC13. Median and interquartile intervals were used to describe HRQoL scores and TTDS. Chi-square tests were performed to assess differences in sociodemographic, clinical characteristics, and incidence of TTD events between chlamydia pneumoniae antibody negative and positive patients. Baseline HRQoL scores of Cpn IgG/IgA negative and positive patients were compared using the Mann–Whitney U Test. Kruskal–Wallis Test was used to compare the baseline HRQoL scores of the combined Cpn IgG and Cpn IgA groups. After controlling for confounding factors, univariate and multivariate Cox regression analyses were used for survival analysis. The results are shown as hazard ratios (HRs) with 95% confidence intervals (CIs). All statistical analyses were performed using R software (version 4.3.0) and Statistical Product and Service Solutions version 20.0 (SPSS version 20.0).

## Results

### Sociodemographic, clinical characteristics, and HRQoL scores at baseline

Between June 2017 and December 2018, a total of 133 serum samples from patients with pathologically diagnosed primary lung cancer were collected and tested for Cpn antibodies. A total of 98 patients completed a baseline questionnaire. Among 98 patients with primary lung cancer, 82 patients completed the first EORTC QLQ-C30 and QLQ-LC13, 50 patients completed the second follow-up, 20 patients completed the third follow-up, 10 patients completed the fourth follow-up, and 2 patients completed the fifth follow-up. In our analysis, all patients (*n* = 82) completed baseline questionnaires and EORTC QLQ-C30 and QLQ-LC13 at least one follow-up. Fifteen patients died during follow-up, with a median follow-up time of 26 months [13,31]. Sixteen patients dropped out during follow-up (dropout rate: 19.5%).

Based on Cpn IgA and Cpn IgG inspection situation, we have three different groups, including the Cpn IgA group (Cpn IgA − , Cpn IgA +), Cpn IgG group (Cpn IgG − , Cpn IgG +), Cpn IgAIgG group (Cpn IgA − IgG − , Cpn IgA + IgG − /IgA − IgG + , Cpn IgA + IgG +). The sociodemographic and clinical characteristics of primary lung cancer patients with different Cpn antibody are shown in Table 1. There were no significant differences in sociodemographic and clinical characteristics between Cpn IgG − and Cpn IgG + patients. There were significant differences in treatment methods between Cpn IgAIgG and Cpn IgA groups (*P* < 0.05), but there were no significant differences in age, body mass index (BMI), education, smoking, alcohol consumption, tissue type, TNM stage, and maximum tumor diameter. In addition, there were significant gender differences between Cpn IgA − and Cpn IgA + patients.

**Table 1:** Characteristics of study patients in demographics and clinical message at baseline

Characteristic	*n* (%)	Groups of Cpn IgA (*n* = 82)		*χ2*	*P*	Groups of Cpn IgG (*n* = 82)		*χ2*	*P*	Groups of Cpn IgAIgG (*n* = 82)			*χ2*	*P*
		Cpn IgA − n (%)	Cpn IgA + n (%)			Cpn IgG − n (%)	Cpn IgG + n (%)			Cpn IgA − IgG − n (%)	Cpn IgA + IgG − /IgA − IgG + n (%)	Cpn IgA + IgG + n (%)		
**Gender**				4.523	**0.033**			0.164	0.686				5.908	0.052
Male	46(56.1)	30 (66.7)	16 (43.2)			12 (60.0)	34 (54.8)			10 (58.8)	22 (71.0)	14 (41.2)		
Female	36(43.9)	15 (33.3)	21 (56.8)			8 (40.0)	28 (45.2)			7 (41.2)	9 (29.0)	20 (58.8)		
**Age**				1.057	0.304			1.042	0.307				0.204	0.903
< = 60	45(54.9)	27 (60.0)	18 (48.6)			9 (45.0)	36 (58.1)			9 (52.9)	18 (58.1)	18 (52.9)		
> 60	37(45.1)	18 (40.0)	19 (51.4)			11 (55.0)	26 (41.9)			8 (47.1)	13 (41.9)	16 (47.1)		
**BMI**					0.925				0.823					0.861
< 18.5	3 (3.7)	2 (4.4)	1 (2.7)			1 (5.0)	2 (3.2)			1 (5.9)	1 (3.2)	1 (2.9)		
[18.5, 24)	48(58.5)	27 (60.0)	21 (56.8)			11 (55.0)	37 (59.7)			9 (52.9)	20 (64.5)	19 (55.9)		
> = 24	31(37.8)	16 (35.6)	15 (40.5)			8 (40.0)	23 (37.1)			7 (41.2)	10 (32.3)	14 (41.2)		
**Educational level**				0.023	0.879			2.726	0.099				2.875	0.238
Primary and below	48(59.3)	27 (60.0)	21 (58.3)			15 (75.0)	33 (54.1)			13 (76.5)	16 (51.6)	19 (57.6)		
Junior high school and above	33(40.7)	18 (40.0)	15 (41.7)			5 (25.0)	28 (45.9)			4 (23.5)	15 (48.4)	14 (42.4)		
**Smoker**				0.912	0.340			0.142	0.706				1.802	0.406
No	44(53.7)	22 (48.9)	22 (59.5)			10 (50.0)	34 (54.8)			9 (52.9)	14 (45.2)	21 (61.8)		
Yes	38(46.3)	23 (51.1)	15 (40.5)			10 (50.0)	28 (45.2)			8 (47.1)	17 (54.8)	13 (38.2)		
**Drinker**				0.931	0.335			2.337	0.126					0.262
No	60(73.2)	31 (68.9)	29 (78.4)			12 (60.0)	48 (77.4)			11 (64.7)	21 (67.7)	28 (82.4)		
Yes	22(26.8)	14 (31.1)	8 (21.6)			8 (40.0)	14 (22.6)			6 (35.3)	10 (32.3)	6 (17.6)		
**Histological type**				0.277	0.599			0.063	0.802					0.656
Non-adenocarcinoma	22(27.2)	13 (29.5)	9 (24.3)			5 (25.0)	17 (27.9)			4 (23.5)	10 (33.3)	8 (23.5)		
Adenocarcinoma	59(72.8)	31 (70.5)	28 (75.7)			15 (75.0)	44 (72.1)			13 (76.5)	20 (66.7)	26 (76.5)		
**TNM stage**				0.647	0.421			1.739	0.187				0.823	0.663
0 and I	47(57.3)	24 (53.3)	23 (62.2)			14 (70.0)	33 (53.2)			11 (64.7)	16 (51.6)	20 (58.8)		
II and above	35(42.7)	21 (46.7)	14 (37.8)			6 (30.0)	29 (46.8)			6 (35.3)	15 (48.4)	14 (41.2)		
**Maximum diameter of tumor**				0.115	0.735			0.163	0.686				0.972	0.615
≤ 2.0	31(40.8)	16 (39.0)	15 (42.9)			7 (36.8)	24 (42.1)			5 (31.2)	13 (46.4)	13 (40.6)		
> 2.0	45(59.2)	25 (61.0)	20 (57.1)			12 (63.2)	33 (57.9)			11 (68.8)	15 (53.6)	19 (59.4)		
**Therapeutic Method**					**0.003**				0.348					**0.009**
Untreated	2 (2.4)	1 (2.2)	1 (2.7)			0 (0.0)	2 (3.2)			0 (0.0)	1 (3.2)	1 (2.9)		
Surgery alone	54(65.9)	23 (51.1)	31 (83.8)			13 (65.0)	41 (66.1)			10 (58.8)	16 (51.6)	28 (82.4)		
Chemotherapy/radiation alone	6 (7.3)	6 (13.3)	0 (0.0)			0 (0.0)	6 (9.7)			0 (0.0)	6 (19.4)	0 (0.0)		
Treated with both chemotherapy/radiation and surgery	20(24.4)	15 (33.3)	5 (13.5)			7 (35.0)	13 (21.0)			7 (41.2)	8 (25.8)	5 (14.7)		

HRQoL baseline scores are expressed in the median and quartile ranges in Tables 2 and 3. Significant differences in Physical functioning (PF) and Social functioning (SF) scale scores were showed between Cpn IgA − and Cpn IgA + groups. Significant differences in the scores of Global health status (QL), Coughing (LC-CO) and Peripheral neuropathy (LC-PN) were showed in the Cpn IgG group. However, there were significant differences in Physical function (PF) and Haemoptysis (LC-HA) in the Cpn IgAIgG group.

**Table 2:** Baseline quality of life scores in Cpn IgA and Cpn IgG groups

	Groups of Cpn IgA (*n* = 82)		*W*	*P*	Groups of Cpn IgG (*n* = 82)		*W*	*P*
	Cpn IgA − (M(P25,P75))	Cpn IgA + (M(P25,P75))			Cpn IgG − (M(P25,P75))	Cpn IgG + (M(P25,P75))		
**QLQ-C30**								
Global health status (QL)	83.33 (66.67, 83.33)	83.33 (66.67, 83.33)	910.5	0.325	66.67 (66.67, 83.33)	83.33 (66.67, 83.33)	386.5	**0.008**
**Functional scales**								
Physical functioning (PF)	93.33 (93.33, 100.00)	93.33 (86.67, 93.33)	1134.0	**0.004**	93.33 (86.67, 100.00)	93.33 (86.67, 100.00)	737.5	0.189
Role functioning (RF)	100.00 (100.00, 100.00)	100.00 (100.00, 100.00)	815.5	0.814	100.00 (100.00, 100.00)	100.00 (100.00, 100.00)	590.0	0.627
Emotional functioning (EF)	91.67 (75.00, 100.00)	83.33 (75.00, 83.33)	1009.5	0.092	87.50 (81.25, 93.75)	83.33 (75.00, 97.92)	703.5	0.359
Cognitive functioning (CF)	100.00 (100.00, 100.00)	100.00 (83.33, 100.00)	887.5	0.482	100.00 (100.00, 100.00)	100.00 (100.00, 100.00)	678.0	0.390
Social functioning (SF)	100.00 (66.67, 100.00)	66.67 (66.67, 100.00)	1082.0	**0.010**	83.33 (66.67, 100.00)	66.67 (66.67, 100.00)	698.0	0.354
**Symptom scales/items**								
Fatigue (FA)	11.11 (0.00, 22.22)	11.11 (0.00, 33.33)	803.5	0.781	22.22 (11.11, 33.33)	5.56 (0.00, 22.22)	781.5	0.068
Nausea and vomiting (NV)	0.00 (0.00, 0.00)	0.00 (0.00, 0.00)	760.0	0.106	0.00 (0.00, 0.00)	0.00 (0.00, 0.00)	610.0	0.805
Pain (PA)	0.00 (0.00, 16.67)	0.00 (0.00, 16.67)	783.5	0.590	8.34 (0.00, 16.67)	0.00 (0.00, 16.67)	750.0	0.095
Dyspnoea (DY)	0.00 (0.00, 33.33)	0.00 (0.00, 33.33)	755.5	0.409	0.00 (0.00, 33.33)	0.00 (0.00, 33.33)	653.0	0.685
Insomnia (SL)	0.00 (0.00, 33.33)	0.00 (0.00, 33.33)	902.0	0.439	0.00 (0.00, 33.33)	0.00 (0.00, 33.33)	732.0	0.147
Appetite loss (AP)	0.00 (0.00, 0.00)	0.00 (0.00, 33.33)	687.0	0.055	0.00 (0.00, 0.00)	0.00 (0.00, 0.00)	608.0	0.860
Constipation (CO)	0.00 (0.00, 0.00)	0.00 (0.00, 0.00)	901.0	0.160	0.00 (0.00, 0.00)	0.00 (0.00, 0.00)	640.0	0.641
Diarrhoea (DI)	0.00 (0.00, 0.00)	0.00 (0.00, 0.00)	810.0	0.281	0.00 (0.00, 0.00)	0.00 (0.00, 0.00)	610.0	0.590
Financial difficulties (FI)	0.00 (0.00, 33.33)	0.00 (0.00, 33.33)	727.5	0.249	0.00 (0.00, 33.33)	0.00 (0.00, 33.33)	587.0	0.678
**QLQ-LC13**								
Dyspnoea (LC-DY)	0.00 (0.00, 11.11)	11.11 (0.00, 22.22)	649.5	0.066	0.00 (0.00, 11.11)	11.11 (0.00, 22.22)	528.0	0.286
Coughing (LC-CO)	33.33 (0.00, 33.33)	33.33 (0.00, 33.33)	829.5	0.980	0.00 (0.00, 33.33)	33.33 (0.00, 66.67)	448.0	**0.047**
Haemoptysis (LC-HA)	0.00 (0.00, 0.00)	0.00 (0.00, 0.00)	938.0	0.057	0.00 (0.00, 0.00)	0.00 (0.00, 0.00)	540.0	0.095
Sore mouth (LC-SM)	0.00 (0.00, 0.00)	0.00 (0.00, 0.00)	869.5	0.203	0.00 (0.00, 0.00)	0.00 (0.00, 0.00)	641.0	0.407
Dysphagia (LC-DS)	0.00 (0.00, 0.00)	0.00 (0.00, 0.00)	851.0	0.378	0.00 (0.00, 0.00)	0.00 (0.00, 0.00)	610.0	0.590
Peripheral neuropathy (LC-PN)	0.00 (0.00, 0.00)	0.00 (0.00, 0.00)	895.5	0.305	0.00 (0.00, 33.33)	0.00 (0.00, 0.00)	764.0	**0.006**
Alopecia (LC-HR)	0.00 (0.00, 0.00)	0.00 (0.00, 0.00)	828.5	0.903	0.00 (0.00, 0.00)	0.00 (0.00, 0.00)	600.0	0.431
Pain in chest (LC-PC)	0.00 (0.00, 33.33)	0.00 (0.00, 33.33)	778.5	0.554	0.00 (0.00, 33.33)	0.00 (0.00, 33.33)	620.5	1.000
Pain in aim or should (LC-PA)	0.00 (0.00, 0.00)	0.00 (0.00, 0.00)	861.5	0.640	0.00 (0.00, 0.00)	0.00 (0.00, 0.00)	564.0	0.291
Pain in other parts (LC-PO)	0.00 (0.00, 0.00)	0.00 (0.00, 0.00)	895.5	0.258	0.00 (0.00, 0.00)	0.00 (0.00, 0.00)	659.5	0.413

**Table 3:** Baseline quality of life scores of patients in the Cpn IgAIgG group

	Groups of Cpn IgAIgG (*n* = 82)			*H*	*P*
	Cpn IgA − IgG − (M(P25,P75))	Cpn IgA + IgG − /IgA − IgG + (M(P25,P75))	Cpn IgA + IgG + (M(P25,P75))		
**QLQ-C30**					
Global health status (QL)	66.67 (66.67, 83.33)	83.33 (66.67, 83.33)	83.33 (66.67, 83.33)	3.040	0.219
**Functional scales**					
Physical functioning (PF)	93.33 (93.33, 100.00)	93.33 (86.67, 100.00)	93.33 (86.67, 93.33)	7.531	**0.023**
Role functioning (RF)	100.00 (100.00, 100.00)	100.00 (91.67, 100.00)	100.00 (100.00, 100.00)	2.830	0.243
Emotional functioning (EF)	91.67 (83.33, 100.00)	83.33 (70.84, 100.00)	83.33 (75.00, 83.33)	3.247	0.197
Cognitive functioning (CF)	100.00 (100.00, 100.00)	100.00 (100.00, 100.00)	100.00 (87.50, 100.00)	1.208	0.547
Social functioning (SF)	100.00 (66.67, 100.00)	100.00 (66.67, 100.00)	66.67 (66.67, 100.00)	5.189	0.075
**Symptom scales/items**					
Fatigue (FA)	11.11 (11.11, 33.33)	11.11 (0.00, 33.33)	11.11 (0.00, 30.55)	0.827	0.661
Nausea and vomiting (NV)	0.00 (0.00, 0.00)	0.00 (0.00, 0.00)	0.00 (0.00, 0.00)	3.920	0.141
Pain (PA)	0.00 (0.00, 16.67)	0.00 (0.00, 16.67)	0.00 (0.00, 16.67)	0.407	0.816
Dyspnoea (DY)	0.00 (0.00, 33.33)	0.00 (0.00, 33.33)	0.00 (0.00, 33.33)	0.129	0.937
Insomnia (SL)	0.00 (0.00, 33.33)	0.00 (0.00, 33.33)	0.00 (0.00, 0.00)	3.981	0.137
Appetite loss (AP)	0.00 (0.00, 0.00)	0.00 (0.00, 0.00)	0.00 (0.00, 33.33)	3.219	0.200
Constipation (CO)	0.00 (0.00, 0.00)	0.00 (0.00, 0.00)	0.00 (0.00, 0.00)	1.589	0.452
Diarrhoea (DI)	0.00 (0.00, 0.00)	0.00 (0.00, 0.00)	0.00 (0.00, 0.00)	1.412	0.494
Financial difficulties (FI)	0.00 (0.00, 33.33)	0.00 (0.00, 33.33)	0.00 (0.00, 33.33)	1.020	0.601
**QLQ-LC13**					
Dyspnoea (LC-DY)	0.00 (0.00, 11.11)	0.00 (0.00, 11.11)	11.11 (0.00, 22.22)	4.002	0.135
Coughing (LC-CO)	0.00 (0.00, 33.33)	33.33 (0.00, 66.67)	33.33 (0.00, 33.33)	4.973	0.083
Haemoptysis (LC-HA)	0.00 (0.00, 0.00)	0.00 (0.00, 0.00)	0.00 (0.00, 0.00)	9.125	**0.010**
Sore mouth (LC-SM)	0.00 (0.00, 0.00)	0.00 (0.00, 0.00)	0.00 (0.00, 0.00)	1.756	0.416
Dysphagia (LC-DS)	0.00 (0.00, 0.00)	0.00 (0.00, 0.00)	0.00 (0.00, 0.00)	1.645	0.439
Peripheral neuropathy (LC-PN)	0.00 (0.00, 33.33)	0.00 (0.00, 0.00)	0.00 (0.00, 0.00)	5.706	0.058
Alopecia (LC-HR)	0.00 (0.00, 0.00)	0.00 (0.00, 0.00)	0.00 (0.00, 0.00)	0.535	0.765
Pain in chest (LC-PC)	0.00 (0.00, 33.33)	0.00 (0.00, 33.33)	0.00 (0.00, 33.33)	0.408	0.816
Pain in aim or should (LC-PA)	0.00 (0.00, 0.00)	0.00 (0.00, 0.00)	0.00 (0.00, 0.00)	1.105	0.575
Pain in other parts (LC-PO)	0.00 (0.00, 0.00)	0.00 (0.00, 0.00)	0.00 (0.00, 0.00)	3.277	0.194

### Time to deterioration and HRQoL events

**Fig. 1:**
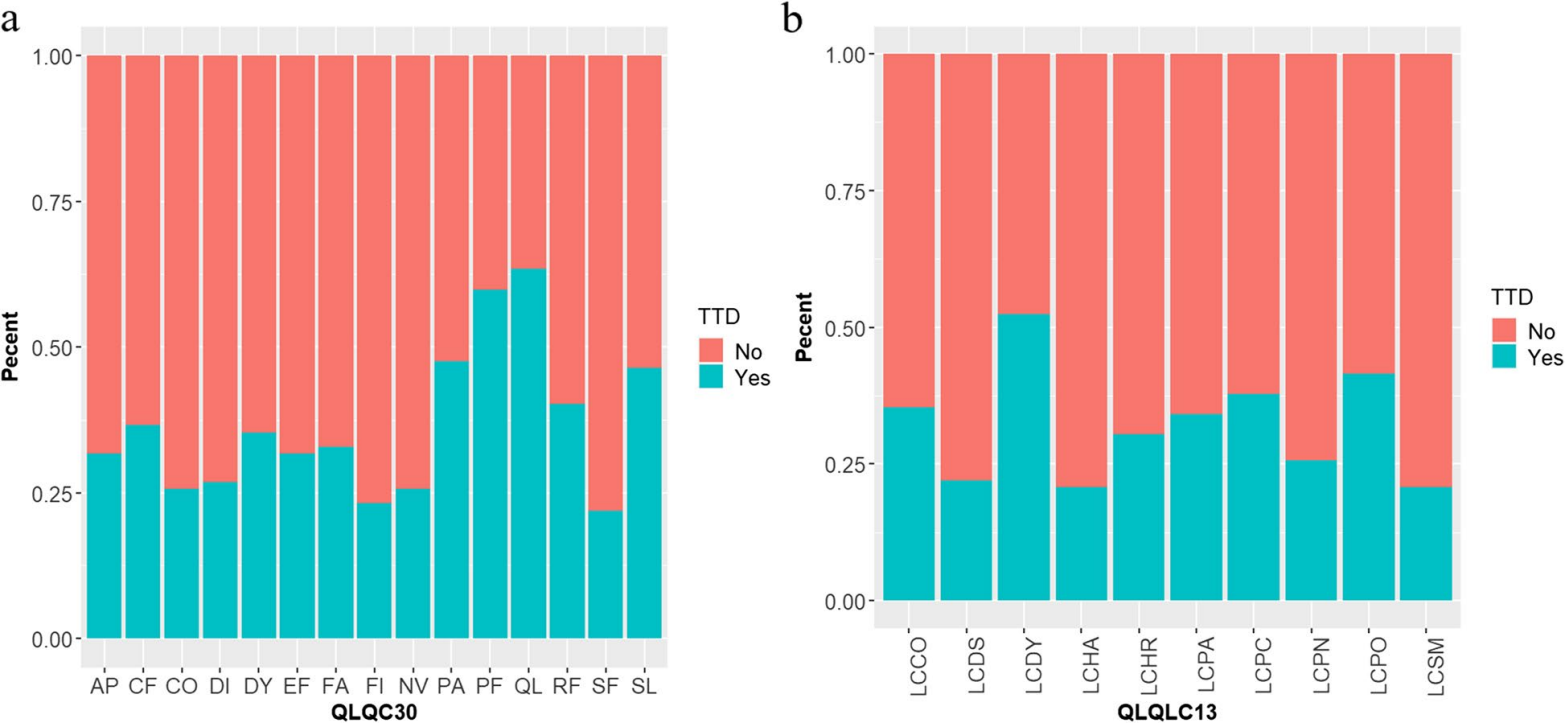
The occurrence of TTD events in EORTC QLQ-C30 (**a**) and EORTC QLQ-LC13 (**b**)

In the EORTC QLQ-C30 functional scale, worsening events of Physical function (PF) were the most common in our cohort during follow-up, while Pain (PA) was the most common in the EORTC QLQ-C30 symptom scale (Fig. 1a). The incidence of dyspnea (LC-DY) TTD events in EORTC QLQ-LC13 was first, and the incidence of Pain in other parts (LC-PO) TTD events was second (Fig. 1b).

### The relationship between TTD and Cpn antibody

As shown in Tables 4, 5 and 6, a significantly higher proportion of patients with Emotional functioning (EF) events in EORTC QLQ-C30 were found in the Cpn IgG − group. A significantly higher proportion of Cpn IgA + IgG − / IgA − IgG + patients showed Physical functioning (PF) events. However, there was no significant difference in TTD events between Cpn IgA − and Cpn IgA + patients.

**Table 4:** Comparison of time to deterioration event in different Cpn IgA status

	Time to deterioration event of Cpn IgAn(%)		*χ2*	*P*	Time to deterioration of Cpn IgAM (P25, P75)		*HR (95% CI)*	*P*
	Cpn IgA −	Cpn IgA +			Cpn IgA −	Cpn IgA +		
**QLQ-C30**								
Global health status (QL)	30 (66.7)	22 (59.5)	0.455	0.500	14.59 (6.11, 27.14)	26.87 (13.37, 30.06)	0.678 (0.385–1.192)	0.177
Functional scales								
Physical functioning (PF)	31 (68.9)	18 (48.6)	3.459	0.063	9.99 (4.57, 23.95)	24.38 (14.55, 29.40)	0.491 (0.270–0.894)	**0.020**
Role functioning (RF)	21 (46.7)	12 (32.4)	1.711	0.191	15.74 (4.67, 29.01)	26.87 (14.78, 30.06)	0.575 (0.275–1.201)	0.141
Emotional functioning (EF)	16 (35.6)	10 (27.0)	0.682	0.409	23.03 (9.99, 30.29)	26.87 (14.78, 30.49)	0.604 (0.267–1.370)	0.228
Cognitive functioning (CF)	17 (37.8)	13 (35.1)	0.061	0.805	23.95 (11.96, 30.29)	26.25 (14.78, 29.73)	0.779 (0.371–1.637)	0.510
Social functioning (SF)	11 (24.4)	7 (18.9)	0.111	0.739	24.05 (11.96, 31.11)	28.02 (16.36, 30.49)	0.658 (0.241–1.798)	0.415
**Symptom scales/items**								
Fatigue (FA)	17 (37.8)	10 (27.0)	1.063	0.303	23.03 (6.60, 29.01)	26.87 (14.55, 30.06)	0.573 (0.254–1.292)	0.179
Nausea and vomiting (NV)	12 (26.7)	9 (24.3)	0.058	0.809	23.95 (12.94, 31.08)	28.85 (16.36, 30.49)	0.725 (0.296–1.778)	0.482
Pain (PA)	24 (53.3)	15 (40.5)	1.332	0.248	12.94 (5.19, 25.95)	24.38 (13.31, 29.70)	0.607 (0.313–1.178)	0.140
Dyspnoea (DY)	19 (42.2)	10 (27.0)	2.051	0.152	23.66 (6.60, 29.70)	28.02 (16.07, 30.49)	0.513 (0.231–1.142)	0.102
Insomnia (SL)	22 (48.9)	16 (43.2)	0.260	0.610	21.19 (7.66, 27.83)	26.87 (14.78, 30.06)	0.656 (0.338–1.274)	0.213
Appetite loss (AP)	15 (33.3)	11 (29.7)	0.122	0.727	23.95 (9.72, 30.29)	28.85 (16.07, 30.49)	0.726 (0.325–1.621)	0.434
Constipation (CO)	13 (28.9)	8 (21.6)	0.563	0.453	23.85 (9.99, 29.70)	28.02 (14.78, 30.49)	0.609 (0.241–1.539)	0.294
Diarrhoea (DI)	15 (33.3)	7 (18.9)	2.149	0.143	23.95 (12.94, 31.08)	28.02 (16.07, 30.49)	0.463 (0.178–1.203)	0.114
Financial difficulties (FI)	11 (24.4)	8 (21.6)	0.091	0.763	23.95 (8.44, 31.08)	28.02 (16.36, 30.49)	0.709 (0.274–1.833)	0.478
**QLQ-LC13**								
Dyspnoea (LC-DY)	26 (57.8)	17 (45.9)	1.140	0.286	12.94 (5.19, 25.95)	25.07 (12.78, 29.40)	0.593 (0.317–1.112)	0.103
Coughing (LC-CO)	18 (40.0)	11 (29.7)	0.937	0.333	18.79 (6.54, 29.01)	28.02 (14.55, 30.49)	0.543 (0.250–1.179)	0.123
Haemoptysis (LC-HA)	10 (22.2)	7 (18.9)	0.135	0.714	23.95 (12.94, 31.08)	28.02 (16.07, 30.49)	0.656 (0.238–1.807)	0.414
Sore mouth (LC-SM)	11 (24.4)	6 (16.2)	0.837	0.360	23.95 (12.94, 31.11)	28.85 (16.36, 30.49)	0.506 (0.175–1.466)	0.210
Dysphagia (LC-DS)	10 (22.2)	8 (21.6)	0.004	0.948	23.95 (12.94, 31.08)	28.02 (16.36, 30.49)	0.772 (0.293–2.033)	0.600
Peripheral neuropathy (LC-PN)	11 (24.4)	10 (27.0)	0.071	0.790	23.95 (12.94, 31.08)	26.25 (14.78, 30.06)	0.951 (0.394–2.296)	0.911
Alopecia (LC-HR)	16 (35.6)	9 (24.3)	1.209	0.272	23.66 (10.28, 29.01)	26.87 (16.07, 30.06)	0.546 (0.232–1.285)	0.166
Pain in chest (LC-PC)	20 (44.4)	11 (29.7)	1.870	0.172	15.74 (7.66, 27.14)	25.95 (14.55, 29.73)	0.479 (0.224–1.026)	0.058
Pain in aim or should (LC-PA)	17 (37.8)	11 (29.7)	0.585	0.444	23.85 (8.67, 29.70)	25.95 (14.78, 29.73)	0.828 (0.379–1.812)	0.637
Pain in other parts (LC-PO)	19 (42.2)	15 (40.5)	0.024	0.878	22.93 (8.80, 28.48)	26.25 (13.37, 29.73)	0.781 (0.390–1.565)	0.486

**Table 5:** Comparison of time to deterioration event in different Cpn IgG status

	Time to deterioration event of Cpn IgGn(%)		*χ2*	*P*	Time to deterioration of Cpn IgGM (P25, P75)		*HR (95% CI)*	*P*
	Cpn IgG −	Cpn IgG +			Cpn IgG −	Cpn IgG +		
**QLQ-C30**								
Global health status (QL)	13 (65.0)	39 (62.9)	0.029	0.866	21.44 (12.97, 30.05)	17.41 (6.50, 29.63)	1.242 (0.648–2.380)	0.514
**Functional scales**								
Physical functioning (PF)	14 (70.0)	35 (56.5)	1.154	0.283	18.09 (6.27, 25.88)	15.15 (5.19, 27.97)	1.126 (0.579–2.190)	0.726
Role functioning (RF)	10 (50.0)	23 (37.1)	1.047	0.306	21.23 (11.36, 30.05)	23.95 (5.20, 29.72)	0.989 (0.454–2.153)	0.977
Emotional functioning (EF)	11 (55.0)	15 (24.2)	6.628	**0.010**	18.71 (11.36, 26.06)	25.95 (13.45, 30.49)	0.422 (0.189–0.941)	**0.035**
Cognitive functioning (CF)	10 (50.0)	20 (32.3)	2.052	0.152	23.76 (14.18, 30.05)	25.63 (13.33, 29.98)	0.701 (0.319–1.544)	0.378
Social functioning (SF)	7 (35.0)	11 (17.7)	1.718	0.190	24.02 (15.45, 31.79)	26.56 (14.61, 30.49)	0.632 (0.232–1.718)	0.368
**Symptom scales/items**								
Fatigue (FA)	8 (40.0)	19 (30.6)	0.599	0.439	23.76 (12.94, 30.05)	24.00 (8.61, 29.72)	0.965 (0.405–2.302)	0.937
Nausea and vomiting (NV)	4 (20.0)	17 (27.4)	0.437	0.509	24.61 (18.03, 34.42)	26.10 (14.61, 30.44)	1.999 (0.585–6.829)	0.269
Pain (PA)	12 (60.0)	27 (43.5)	1.641	0.200	18.71 (8.93, 29.18)	17.41 (5.44, 28.85)	0.900 (0.443–1.831)	0.771
Dyspnoea (DY)	9 (45.0)	20 (32.3)	1.074	0.300	24.12 (14.18, 31.79)	25.19 (10.06, 29.98)	0.914 (0.400–2.088)	0.831
Insomnia (SL)	12 (60.0)	26 (741.9)	1.985	0.159	20.86 (8.94, 30.75)	24.56 (10.41, 29.63)	0.769 (0.378–1.565)	0.469
Appetite loss (AP)	7 (35.0)	19 (30.6)	0.132	0.716	24.02 (14.18, 31.79)	25.95 (13.67, 30.44)	1.077 (0.429–2.704)	0.875
Constipation (CO)	7 (35.0)	14 (22.6)	1.224	0.269	23.76 (14.18, 30.05)	25.63 (13.33, 30.23)	0.771 (0.296–2.010)	0.595
Diarrhoea (DI)	5 (25.0)	17 (27.4)	0.045	0.832	24.12 (15.45, 31.79)	26.10 (13.67, 30.44)	1.387 (0.466–4.128)	0.557
Financial difficulties (FI)	7 (35.0)	12 (19.4)	1.293	0.256	21.23 (11.36, 30.05)	26.56 (14.61, 30.49)	0.605 (0.227–1.613)	0.315
**QLQ-LC13**								
Dyspnoea (LC-DY)	13 (65.0)	30 (48.4)	1.673	0.196	15.79 (6,25, 29.18)	20.75 (7.85, 28.85)	0.865 (0.438–1.707)	0.675
Coughing (LC-CO)	8 (40.0)	21 (33.9)	0.249	0.618	24.02 (8.93, 32.30)	24.00 (8.50, 29.72)	0.965 (0.410–2.271)	0.935
Haemoptysis (LC-HA)	5 (25.0)	12 (19.4)	0.050	0.823	24.12 (15.45, 31.79)	26.10 (13.67, 30.44)	0.973 (0.313–3.019)	0.962
Sore mouth (LC-SM)	5 (25.0)	12 (19.4)	0.050	0.823	24.61 (18.03, 34.42)	26.10 (14.61, 30.49)	1.086 (0.349–3.383)	0.887
Dysphagia (LC-DS)	6 (30.0)	12 (19.4)	0.475	0.491	23.76 (15.45, 30.05)	26.56 (15.10, 30.49)	0.737 (0.259–2.096)	0.567
Peripheral neuropathy (LC-PN)	7 (35.0)	14 (22.6)	1.224	0.269	23.76 (15.45, 30.05)	25.95 (13.33, 30.44)	0.746 (0.286–1.945)	0.549
Alopecia (LC-HR)	9 (45.0)	16 (25.8)	2.629	0.105	23.30 (13.11, 29.18)	25.63 (13.33, 29.98)	0.617 (0.264–1.444)	0.266
Pain in chest (LC-PC)	9 (45.0)	22 (35.5)	0.582	0.445	20.86 (12.94, 29.18)	22.11 (8.27, 28.99)	0.985 (0.439–2.214)	0.972
Pain in aim or should (LC-PA)	9 (45.0)	19 (30.6)	1.386	0.239	23.30 (15.45, 29.18)	25.19 (10.02, 29.98)	0.635 (0.285–1.417)	0.267
Pain in other parts (LC-PO)	12 (60.0)	22 (35.5)	3.745	0.053	23.30 (14.18, 29.18)	23.95 (8.78, 29.63)	0.723 (0.348–1.503)	0.385

**Table 6:** Comparison of time to deterioration event in different Cpn IgAIgG status

	Time to deterioration event of Cpn IgAIgGn(%)			*χ2*	*P*	Time to deterioration of Cpn IgAIgGM (P25, P75)			*HR (95% CI)*	*P*
	Cpn IgA-IgG-	Cpn IgA + IgG-/IgA-IgG +	Cpn IgA + IgG +			Cpn IgA-IgG-	Cpn IgA + IgG-/IgA-IgG +	Cpn IgA + IgG +		
**QLQ-C30**										
Global health status (QL)	10 (58.8)	23 (74.2)	19 (55.9)	2.538	0.281	19.94 (12.94, 29.70)	11.96 (4.54, 26.55)	27.45 (13.33, 29.98)	0.916 (0.645–1.300)	0.623
**Functional scales**										
Physical functioning (PF)	11 (64.7)	23 (74.2)	15 (44.1)	6.318	**0.042**	15.74 (5.45, 24.84)	9.99 (4.62, 23.95)	24.81 (13.67, 29.31)	0.786 (0.550–1.123)	0.186
Role functioning (RF)	7 (41.2)	17 (54.8)	9 (26.5)	5.434	0.066	18.79 (6.60, 29.70)	18.63 (4.62, 28.16)	27.45 (14.61, 29.98)	0.802 (0.515–1.249)	0.328
Emotional functioning (EF)	9 (52.9)	9 (29.0)	8 (23.5)	4.692	0.096	15.74 (6.60, 24.84)	24.38 (11.12, 31.85)	27.45 (14.61, 30.38)	0.591 (0.354–0.988)	**0.045**
Cognitive functioning (CF)	8 (47.1)	11 (35.5)	11 (32.4)	1.083	0.582	23.66 (12.94, 29.70)	24.05 (12.57, 30.70)	26.56 (14.61, 29.72)	0.800 (0.500–1.279)	0.351
Social functioning (SF)	5 (29.4)	8 (25.8)	5 (14.7)		0.399	23.66 (14.59, 31.08)	24.38 (11.12, 31.85)	28.44 (16.14, 30.38)	0.717 (0.387–1.328)	0.290
**Symptom scales/items**										
Fatigue (FA)	7 (41.2)	11 (35.5)	9 (26.5)	1.257	0.533	18.79 (12.94, 29.01)	23.95 (6.12, 30.70)	26.56 (13.67, 29.72)	0.790 (0.481–1.296)	0.350
Nausea and vomiting (NV)	3 (17.6)	10 (32.3)	8 (23.5)		0.549	23.85 (15.74, 31.08)	24.38 (11.12, 31.85)	28.44 (16.14, 30.38)	1.056 (0.593–1.879)	0.853
Pain (PA)	10 (58.8)	16 (51.6)	13 (38.2)	2.254	0.324	14.59 (6.54, 29.01)	10.28 (5.19, 25.63)	24.96 (12.91, 29.63)	0.791 (0.527–1.187)	0.257
Dyspnoea (DY)	8 (47.1)	12 (38.7)	9 (26.5)	2.345	0.310	23.66 (12.94, 29.70)	23.95 (7.00, 30.70)	27.45 (15.10, 29.98)	0.745 (0.462–1.201)	0.226
Insomnia (SL)	11 (64.7)	12 (38.7)	15 (44.1)	3.099	0.212	15.47 (6.60, 24.84)	23.95 (9.22, 29.39)	26.56 (14.78, 29.72)	0.761 (0.498–1.162)	0.206
Appetite loss (AP)	6 (35.3)	10 (32.3)	10 (29.4)	0.188	0.910	22.93 (12.94, 29.70)	24.38 (9.66, 31.85)	28.44 (15.10, 30.38)	0.894 (0.535–1.494)	0.669
Constipation (CO)	6 (35.3)	8 (25.8)	7 (20.6)		0.519	22.93 (12.94, 29.01)	24.38 (9.66, 31.85)	27.45 (14.61, 29.98)	0.743 (0.419–1.321)	0.312
Diarrhoea (DI)	4 (23.5)	12 (38.7)	6 (17.6)		0.157	23.66 (14.59, 29.70)	25.30 (11.73, 35.09)	27.45 (15.10, 29.98)	0.801 (0.449–1.430)	0.453
Financial difficulties (FI)	5 (29.4)	8 (25.8)	6 (17.6)		0.624	18.79 (6.60, 29.70)	24.38 (10.14, 31.85)	28.44 (16.14, 30.38)	0.728 (0.400–1.324)	0.298
**QLQ-LC13**										
Dyspnoea (LC-DY)	11 (64.7)	17 (54.8)	15 (44.1)	2.041	0.360	12.94 (5.39, 29.01)	14.36 (5.19, 25.17)	25.51 (9.95, 29.31)	0.769 (0.521–1.135)	0.185
Coughing (LC-CO)	7 (41.2)	12 (38.7)	10 (29.4)	0.930	0.628	18.79 (6.54, 29.70)	23.95 (8.05, 29.39)	27.45 (13.67, 30.30)	0.773 (0.482–1.239)	0.285
Haemoptysis (LC-HA)	4 (23.5)	7 (22.6)	6 (17.6)		0.826	23.66 (14.59, 29.70)	25.30 (11.73, 35.09)	27.45 (15.10, 29.98)	0.832 (0.441–1.569)	0.570
Sore mouth (LC-SM)	4 (23.5)	8 (25.8)	5 (14.7)		0.466	23.85 (15.74, 31.08)	24.38 (11.12, 35.09)	28.44 (16.14, 30.38)	0.785 (0.418–1.476)	0.453
Dysphagia (LC-DS)	4 (23.5)	8 (25.8)	6 (17.6)		0.738	23.66 (14.59, 29.70)	24.38 (15.21, 31.85)	28.44 (16.14, 30.38)	0.810 (0.438–1.500)	0.503
Peripheral neuropathy (LC-PN)	5 (29.4)	8 (25.8)	8 (23.5)		0.946	23.66 (14.59, 29.70)	24.38 (15.21, 31.85)	26.56 (13.72, 29.98)	0.886 (0.501–1.569)	0.679
Alopecia (LC-HR)	7 (41.2)	11 (35.5)	7 (20.6)	2.854	0.240	22.93 (12.94, 29.01)	23.95 (10.53, 29.39)	27.45 (15.10, 29.98)	0.661 (0.393–1.111)	0.118
Pain in chest (LC-PC)	7 (41.2)	15 (48.4)	9 (26.5)	3.416	0.181	18.79 (12.94, 29.01)	18.46 (6.89, 26.55)	26.41 (13.67, 29.72)	0.756 (0.486–1.176)	0.214
Pain in aim or should (LC-PA)	7 (41.2)	12 (38.7)	9 (26.5)	1.552	0.460	23.66 (14.59, 29.70)	23.95 (9.00, 28.16)	27.45 (13.72, 29.98)	0.793 (0.487–1.290)	0.350
Pain in other parts (LC-PO)	9 (52.9)	13 (41.9)	12 (35.3)	1.459	0.482	22.93 (12.94, 29.01)	23.03 (8.56, 28.16)	26.56 (13.33, 29.72)	0.811 (0.525–1.251)	0.343

In univariate Cox regression analysis, Cpn IgA + was associated with improved HRQoL in Physical functioning (PF) (*HR* = 0.491, *95% CI*: 0.270–0.894, *P* = 0.020). Cpn IgG + and Cpn IgA + IgG − /IgA − IgG + (*HR* = 0.591, *95% CI*:0.354–0.988, *P* = 0.045) indicate Emotional functioning (EF) (*HR* = 0.422, *95% CI*: 0.189–0.941, *P* = 0.035).

To minimize the impact of potential confounding factors, we adjusted for baseline variables (including sex and smoking) that were significant in the univariate Cox regression analysis of the Cpn IgA group. No significant variables were found for Cpn IgG group in univariate Cox regression, so we adjusted for all baseline variables (including age, sex, BMI, education, smoking and alcohol consumption) and clinical variables (including tissue type, TNM stage, maximum tumor diameter, and treatment) in our multivariate Cox regression analysis. The results were similar to those obtained by single-factor Cox regression analysis. Cpn IgA was associated with shorter time to deterioration of Physical functioning (PF) (*HR* = 0.539, *95% CI*: 0.291–0.996, *P* = 0.048), while Cpn IgG was associated with shorter time to deterioration in Emotional functioning (EF) (*HR* = 0.310, *95% CI*: 0.115–0.836, *P* = 0.021) (Table 7).

**Table 7:** Multivariate Cox analysis for time to deterioration event ≥ 5 points

Variable	Items	HR (95%CI)	P
**QLQ-C30**			
Cpn IgA	Physical functioning (PF)	0.539 (0.291–0.996)	**0.048**
Cpn IgG	Emotional functioning (EF)	0.310 (0.115–0.836)	**0.021**

## Discussion

As lung cancer patients live longer, it is increasingly important to improve health-related quality of life. Previous studies have found that chronic Cpn infection may be closely related to the occurrence and development of lung cancer [20, 21]. Therefore, we have reason to believe that chronic Cpn infection may affect the prognostic quality of life of lung cancer patients. In this study, we constructed a TTD model of primary lung cancer containing EORTC QLQ-C30 and QLQ-LC13 in a prospective study. We found that the presence of Cpn antibodies prior to treatment affected TTD in Physical functioning and Emotional functioning.

In the functional scale of EORTC QLQ-C30 in this study, Physical function (PF) TTD events were the most common, while Pain (PA) was the most common on the symptom scale. The incidence of dyspnea (LC-DY)TTD events in EORTC QLQ-LC13 was the first, and that of Pain in other parts (LC-PO) was the second. However, we found that Cpn antibodies only affected TTD events in Physical functioning (PF) and Emotional functioning (EF). These findings suggest that Physical functioning (PF) and Emotional functioning (EF) deserve more clinical attention.

In recent years, studies have reported that pulmonary inflammatory diseases are significantly associated with the risk of lung cancer. Chlamydia pneumoniae is closely related to chronic lung inflammation and may play an important role in the progression of lung cancer [22]. It has been found that IgA antibodies were increased in lung cancer patients infected with Chlamydia pneumoniae [23]. In another study, increased Chlamydia pneumoniae-specific IgA levels in smokers with lung cancer were found [24]. A mata analysis that included 13 studies, 2553 lung cancer cases and 2460 controls showed that chlamydia pneumoniae infection was significantly associated with the risk of lung cancer, with IgA infection having a 3.19 times greater risk than negative titers (95% CI: 1.96–5.19), the risk of IgG infection was 2.02 times that of negative titers (95% CI: 1.29–3.16) [25]. To verify the relationship between Mycoplasma pneumoniae and lung cancer, more research work is needed to gain insight into the relationship between Cpn infection and primary lung cancer, and to develop more effective prevention and treatment strategies to improve the quality of life of lung cancer patients.To our knowledge, this is the first prospective study to explore the relationship between Cpn infection and HRQoL based on the TTD model. Our results may provide a new perspective for improving the quality of life of patients with primary lung cancer. Still, there are some limitations to our study. First, in this study, we did not evaluate the effect of Cpn antibody titer level on HRQoL. Secondly, 16 patients dropped out of our study, possibly due to disease progression or deterioration within a short time after treatment, or due to lack of follow-up. Therefore, there will inevitably be some subsequent bias in our study, leading to bias in the association estimation of exposure results. In addition, due to the small study sample size, Cpn infection may affect the judgment of HRQoL in patients with primary lung cancer. It is necessary to expand the sample size and extend the follow-up time to further explore the relationship between Cpn infection and HRQoL in primary lung cancer.

## Conclusions

According to EORTC QLQ-C30 and EORTC QLQ-LC13, positive Cpn IgA delayed TTD in Physical functioning and Cpn IgG delayed TTD in Emotional functioning. Our report enables us to hypothesize that pretreatment Cpn infection may affect HRQoL in patients with primary lung cancer.

## Data Availability

The datasets used or analysed during the current study are available from the corresponding author on reasonable request.

## Abbreviations

Cpn: Chlamydia pneumoniae
TTD: Time to deterioration
HRQoL: Health-related Quality of Life
EORTC QLQ-C30: EORTC Quality of Life Questionnaire
EORTC QLQ-LC13: EORTC Quality of Life Questionnaire-Lung Cancer
PF: Physical function
PA: Pain
LC-DY: Dyspnea
QoL: Quality of life
HRs: Hazard ratios
CIs: Confidence intervals
BMI: Body mass index
SF: Social functioning
QL: Global health status
LC-CO: Coughing
LC-PN: Peripheral neuropathy
LC-HA: Haemoptysis
LC-PO: Pain in other parts
EF: Emotional functioning
